# Utilizing sequence intrinsic composition to classify protein-coding and long non-coding transcripts

**DOI:** 10.1093/nar/gkt646

**Published:** 2013-07-27

**Authors:** Liang Sun, Haitao Luo, Dechao Bu, Guoguang Zhao, Kuntao Yu, Changhai Zhang, Yuanning Liu, Runsheng Chen, Yi Zhao

**Affiliations:** ^1^Bioinformatics Research Group, Advanced Computing Research Laboratory, Institute of Computing Technology, Chinese Academy of Sciences, Beijing 100190, China, ^2^College of Computer Science and Technology, Jilin University, Changchun 130012, China and ^3^Laboratory of Bioinformatics and Non-coding RNA, Institute of Biophysics, Chinese Academy of Sciences, Beijing 100101, China

## Abstract

It is a challenge to classify protein-coding or non-coding transcripts, especially those re-constructed from high-throughput sequencing data of poorly annotated species. This study developed and evaluated a powerful signature tool, Coding-Non-Coding Index (CNCI), by profiling adjoining nucleotide triplets to effectively distinguish protein-coding and non-coding sequences independent of known annotations. CNCI is effective for classifying incomplete transcripts and sense–antisense pairs. The implementation of CNCI offered highly accurate classification of transcripts assembled from whole-transcriptome sequencing data in a cross-species manner, that demonstrated gene evolutionary divergence between vertebrates, and invertebrates, or between plants, and provided a long non-coding RNA catalog of orangutan. CNCI software is available at http://www.bioinfo.org/software/cnci.

## INTRODUCTION

The recent progress in the ENCODE project suggests that >70% of human genome sequences are transcribed into processed or primary RNAs (1,2). For other species, numerous novel transcripts are identified by advances in RNA sequencing techniques (RNA-seq) (3). It remains a challenge to identify sequence differences between protein-coding and non-coding transcripts. During the past 5 years, several tools, such as CPC and phyloCSF, have been developed to classify protein-coding or non-coding transcripts using information on open reading frame (ORF), known protein database, or evolutionary signatures (4–6). These approaches have been effective in identifying long coding transcripts for putative ORF or peptide hits. However, they are not suitable for identifying long non-coding RNAs (lncRNAs), which may contain short protein-like sub-sequences or long putative ORFs (6,7). Moreover, these available tools search for the best segment of evolutionary signatures but may also lead to significant false-positive and false-negative discoveries, because most of the functional non-coding RNAs have higher evolutionary conservation relative to introns, which suggested the conserved element exists in these non-coding sequences (8), and in contrast, many newly evolved proteins do not contain a conserved ORF (7). In addition, for poorly annotated species or those without whole-genome sequence, it is hard to define the transcript status using the existing tools.

To overcome these challenges, we developed Coding-Non-Coding Index (CNCI) software, a powerful signature tool, by profiling adjoining nucleotide triplets (ANT), to effectively distinguish between protein-coding and non-coding sequences independent of known annotations. In comparison with the existing tools, CNCI showed better performance in many aspects, especially for classification of incomplete transcripts and sense–antisense transcript pairs. Notably, CNCI performed well uniformly on all the species of the vertebrates, but relatively poorly for invertebrates and plants, when using human data as training sets. Because CNCI can classify protein-coding and non-coding RNAs solely based on sequence intrinsic composition, it is potentially applicable to a variety of species without whole-genome sequence or with poorly annotated information. As an example of application for poorly annotated species, we tested CNCI on a published RNA-Seq data set from six organs of orangutan. As a result, CNCI annotated 7697 novel transcripts as lncRNAs, which contributed to the first comprehensive orangutan lncRNA catalog.

## MATERIALS AND METHODS

### Data description

For human training sets, protein-coding genes were collected from RefSeq database and long non-coding genes were collected from Gencode (v11) (9). For mouse testing sets, both protein-coding and non-coding genes were collected from Ensembl (v65) (10) database. As other testing sets, gene annotation of other vertebrates or plants was downloaded from Ensembl (v69) and EnsemblPlants (v16) (10) databases, respectively. LincRNA catalog was obtained from human body map (11). Whole-transcriptome sequencing data of the six organs of orangutan were obtained from the study of David Brawand *et al.* (3) and downloaded from Gene Expression Omnibus under accession code GSE30352. All the data were summarized in Supplementary Table S1, and the length distributions of the human and mouse transcript collections are depicted in Supplementary Table S2.

### Calculation of usage frequency of ANT

In this study, we began analyzing the usage frequency of ANT in coding domain sequence (CDS) and non-coding RNA sequences. There were 64*64 ANT, and we calculated usage frequency of each ANT as follows:

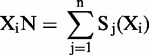


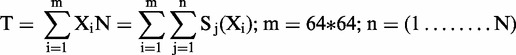


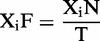

**Figure 1.**
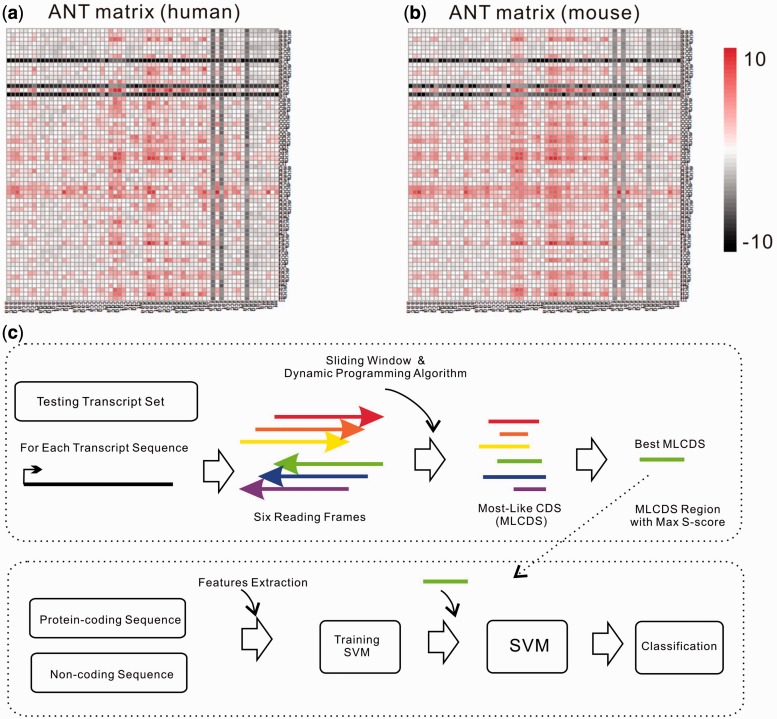
Illustration of ANT score matrix and CNCI framework. The score of each ANT is calculated for human (**a**) or mouse (**b**). The three black rows or columns represent three stop codons, including UAA, UAG and UGA (corresponding to ATT, ATC and ACT in cDNA sequence, respectively), which shows low frequency in protein-coding sequence. (**c**) The framework of CNCI. The top panel shows the process of a sequence in a testing set. For a given sequence, six MLCDS regions (represented by six lines) are identified from six reading frames (represented by six color arrow lines) using a sliding window and dynamic programming algorithm. Then, an MLCDS region with a maximal S-score is selected to incorporate into an SVM. The bottom panel shows the training and classification process. Reliable protein-coding and non-coding sequences are used as a training set, and five features are extracted to train SVM, which classifies the incorporating sequence into protein-coding or non-coding sequence.

Where X indicates the ANT, S_j_(X_i_) is the occurrence number of X in sequence i, X_i_N is X_i_’s total occurrence number in the data set, T is all kinds of ANT’s total occurrence number in the data set and X_i_F is the usage frequency of the ANT. In human sequences, the usage frequency of ANT was calculated in 30 507 CDS and 18 566 long non-coding transcripts. In mouse sequences, the usage frequency was calculated in 25 316 CDS and 8696 long non-coding transcripts (Supplementary Figure S1). The log-ratio of the usage frequency of all kinds of ANT constituted a 64*64 ANT score matrix (Figure 1a and b).

### Utilization of the sliding window to scan each transcript

It is an essential step for our approach to identify the most-like CDS (MLCDS) region of each transcript. We first used the sliding window to analyze each transcript by setting the size of the sliding window and the scan step as one ANT. To verify the size of the sliding window (represented by parameter N) to achieve the robust final classification result, we defined a series of N’s with different lengths (from 30 nt to 300 nt, with 30 nt as a step-forward interval). For each of the N sequences, a classifier was trained with human training data. The sensitivity–specificity curves of the classification were then calculated on the testing set (Supplementary Figure S2). In our classification model, N of 150 nt was found to be the robust, and thus we chose *N* = 150 nt, which is a proper size longer than small RNAs but shorter than lncRNAs, into the classification model.

The window scanned each transcript six times to generate six reading frames. At the same time, CNCI calculated the sequence-score (S-score) of each window based on ANT score matrix; thus, a given transcript produced six discrete numerical arrays. In the process of sliding window, each transcript was converted into six arrays, and each array was composed of sliding window’s S-score of one kind of reading frame. S-score can reflect the coding ability of each sliding window, and our initial purpose was to find out sub-sequences (described as MLCDS earlier in the text) in each of the six reading frames that have the most ability to code.

### Prediction of the MLCDS in each of the six reading frames

To identify the MLCDS of each reading frame, we applied a dynamic programming called Maximum Interval Sum (12). This method can scan one numerical array (which contains positive and negative numbers), and identify a consecutive sub-array, which has the largest sum value than any other sub-arrays even including the whole array, using the following formula:

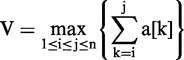

Where V is the maximum interval sum in a reading frame, a[k] is the maximum substring corresponding to V and i and j represent the start and end position of a[k] in this reading frame, respectively. To calculate V, i and j, we introduced a limited local maximum interval sum, b[j], which is defined as:

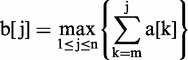

Where m is a variable representing the start position of b[j], j is the end position of b[j] and b[j] is the local maximum interval sum. By combining with the definition of V, we can deduce that:

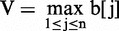



This formula means that V is the maximum value of b[j] sets. According to the definition of b[j], we can draw a conclusion that when b[j − 1] > 0, b[j] = b[j − 1] + a[j] whatever a[j] is. When b[j − 1] < 0, b[j] = a[j] whatever a[j] is. Thus, we can use the dynamic programming to scan the numerical array of one reading frame, as stated in the following rule:

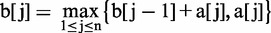



After the aforementioned processes, six candidate MLCDS regions from the six reading frames of each transcript were derived, and the maximum one as the best MLCDS region of the transcript that had a significant larger length and higher quality percentage than the other five candidates was used to perform the feature extraction. To estimate the quality of the MLCDS, we compared the MLCDS of all human protein-coding transcripts with the corresponding true CDS and evaluated the distribution of overlap degree (Supplementary Figure S3).

### Feature extraction and classification model construction

To distinguish protein-coding sequences from the non-coding sequences, we extracted five features, i.e. the length and S-score of MLCDS, length-percentage, score-distance and codon-bias. The length and S-score of MLCDS were used as the first two features, which assess the extent and quality of the MLCDS, respectively (Supplementary Table S3). Moreover, as demonstrated earlier in the text, protein-coding transcripts possess a special reading frame obviously distinct from the other five in the distribution of ANT. We analyzed six MLCDS candidates outputted by dynamic programming of the six reading frames for each transcript, with the assumption that there must exist one best MLCDS (as described earlier in the text); however, this phenomenon does not generally exist for non-coding transcripts. Thus, we defined other two features, length-percentage and score-distance, as follows:

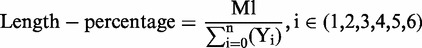

Where Ml is the length of the best MLCDS (according to S-score value) among that of six reading frames, and Y_i_ represents the length of each six of the MLCDS.

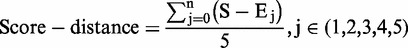

Where S is the S-score of the best MLCDS, and Ej represents the S-score of the other five MLCDS (Supplementary Table S3).

All aforementioned four selected features could, to some extent, distinguish the protein-coding and non-coding sequences and were concordantly higher in protein-coding transcripts and lower in non-coding transcripts (Supplementary Figure S4). Finally, we included the fifth feature, the frequency of single nucleotide triplets, in the MLCDS as the last feature to complement the construction of a classification model. This feature was defined as codon-bias, which evaluated the coding-non-coding bias for each of the 61 kinds of codons (the three stop codons were ruled out) (Supplementary Figure S5).

To get the positive and negative training sets, we extracted the five features for each best MLCDS from the known protein-coding and non-coding transcript data sets, respectively. We then incorporated these two training sets into a support vector machine (SVM) as a model construction (Figure 1c). We used the A Library for Support Vector Machines (LIBSVM) (13) to train an SVM model using the standard radial basis function kernel, where the C and gamma parameters were set by default.

### Identification of orangutan lncRNAs

To identify orangutan lncRNAs, we used the spliced read aligner TopHat (14) (version V1.3.1) to map all sequenced reads to the orangutan genome (ponAbe2) with the following parameters: min-anchor = 5, min-isoform-fraction = 0 and the rest set as default. We then aligned reads of each tissue from TopHat and assembled them into transcriptome separately by Cufflinks (15) (version 1.0.3) with default parameters (and ‘min-frags-per-transfrag = 0’). After that, we constructed the transcripts from six tissues and merged them together to constitute the final transcript set of orangutan and then compared them with known genes annotated by Ensembl database (v69). Novel long transcripts (>200 bp) that do not overlap with any known annotation and are localized in intronic, antisense or intergenic region were filtered by CNCI and added to the lncRNA catalog of orangutan.

## RESULTS

### Overview of CNCI methodology

CNCI contains two main steps, including scoring the sequence and construction of classification model. To use ANT signature to classify protein-coding and non-coding sequences, we constructed ANT score matrix that represents the degree of coding-non-coding bias. First, we calculated the usage frequency of ANT in true CDS and non-coding sequences separately (Supplementary Figure S1) and the log-ratio of the usage frequency between coding and non-coding sequences for each ANT. For human sequences as example, the usage frequency of ANT was calculated in 30 507 CDS and 18 566 long non-coding transcripts. Then, the log-ratio of the usage frequency of all kinds of ANT constituted a 64*64 ANT score matrix (Figure 1a and b). According to this matrix, S-score of six reading frames for each sequence was calculated using the following formula:

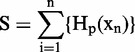

Where S is the S-score, H_p_ is the ANT score matrix, X represents the types of ANT and n is the length of sequences in nucleotide triplet format.

We next evaluated whether S-score is effective in classifying coding and non-coding sequences. First, we compared the S-score distribution of true CDS with that of other five reading frames of human protein-coding transcripts, which revealed a distinct pattern, where the true CDS frame had the highest score among all six frames (Supplementary Figure S6). Then, we normalized the length of true CDS and all other five reading frames of all coding transcripts and plotted the ANT score for each position. The data showed the same pattern with distribution of S-score (Supplementary Figure S7). For non-coding transcripts, the ANT scores across the normalized length showed a pattern similar to those of the other five non-coding reading frames of protein-coding transcripts (Supplementary Figure S7). The results confirmed the classification ability of S-score and suggested that it is crucial to first identify MLCDS region and then classify any given sequences according to the best MLCDS using these six reading frames of a coming sequence regardless of whether it is an actual protein-coding transcript or not.

To construct a classification model, we used a sliding window approach to compute S-score for each reading frame of a sequence in each of the windows. Then, we identified the MLCDS for each reading frame according to the cumulative S-score of the combined windows using a dynamic programming algorithm. Subsequently, we extracted these five statistical features from the best MLCDS that have a maximal S-score among these six reading frames and incorporated them into an SVM classifier to train this classification model (Figure 1c).

### CNCI performance and comparison with existing tools

**Figure 2.**
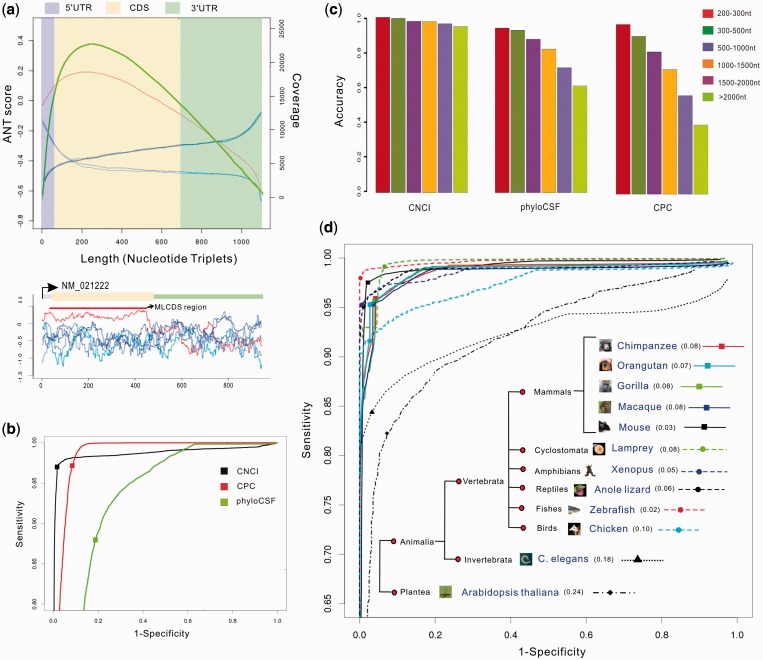
CNCI performance. (**a**) The top panel shows ANT score distribution (the left *y*-axis) of these six reading frames for each protein-coding transcript, whose length is normalized to 1100 nucleotide triplets in the *x*-axis. Red line represents the correct transcriptional reading frame and other five lines (blue or green) represent other five reading frames. Green line indicates the distribution of the coverage (the right *y*-axis) of the MLCDS region for each protein-coding transcript across the normalized length. The three regions marked by blue, yellow and green indicate the mean length of 3′UTR (6%), CDS (56.6%) and 5′UTR (37.4%), respectively, across the normalized length. The bottom panel shows an example of a gene NM_021222. (**b**) The ROC analyses of CNCI, CPC and phyloCSF. The MAE denoted by solid squares is 0.05, 0.11 and 0.28, respectively. (**c**) The accuracy of CNCI, CPC and phyloCSF for classification of different lincRNA lengths. (**d**) The ROC curves and taxonomic tree of 12 species. The minimum error rate is marked following the name of species.

We applied CNCI to reliable protein-coding and non-coding data sets of both human and mouse and assessed its performance in the case of cross-species. We first trained the CNCI on human protein-coding and long non-coding transcripts that showed 97.3% accuracy by 10-fold cross-validation. For all protein-coding transcripts, the correct transcriptional reading frame showed a notable peak of the ANT scores within the CDS region, and had distinct pattern compared with the other five reading frames (Figure 2a). The coverage of the maximum MLCDS for all the protein-coding transcripts was consistent with the distribution of the ANT scores (Figure 2a), and a high degree of coincidence between MLCDS and CDS was observed (Supplementary Figure S3). However, these phenomena did not occur in non-coding transcripts, suggesting that the CNCI method has robust strength (Supplementary Figure S7). Next, we applied the learned regularities to classify objects in a test set, which was collected from mouse protein-coding and non-coding transcripts. We found that the minimum average error (MAE) (the cutoff that minimizes the average false-positive and false-negative rates) was 0.05 after the examination of the receiver operating characteristic (ROC) curve (Figure 2b). The result showed that CNCI worked reasonably well on mouse data, although CNCI was trained on human sequences. Moreover, we also compared the performance of CNCI with that of other available tools by re-analyzing the test data set using CPC and phyloCSF. The ROC curves showed that MAE of CNCI was lower than that of other two methods (MAE was 0.11 and 0.28 for CPC and phyloCSF, respectively), indicating that CNCI is a better tool (Figure 2b). In addition, we further tested CNCI as well as CPC and phyloCSF, on an independent long intergenic non-coding RNA data set from human body map lincRNAs catalog (11). After removing the overlapping transcripts with training set, we examined their performance across different lengths of transcripts, and found that CNCI had better performance on all non-coding transcripts with various lengths, whereas CPC and phyloCSF had poor performance on transcripts with longer sequences (Figure 2c).

Because both the reference and the reconstructed transcripts in RNA-Seq experiments may be incomplete, we modified known gene annotation by trimming the exon at 3′- or 5′-end of each transcript to generate a modified transcript data set (to mimic the incomplete RNA-Seq data) and re-evaluated CNCI performance in these incomplete transcripts. There were 28.3 and 45.2% of known protein-coding transcripts with a complete CDS after trimming the 5′ and 3′ exon, respectively (Supplementary Table S4). CNCI maintained its high accuracy in the modified transcript data sets with a mean accuracy of 97.9 and 97.7%, respectively, which was higher than that of CPC (87.1 and 87.9%, respectively) and phyloCSF (82.0 and 82.3%, respectively) (Supplementary Table S5). To address whether CNCI is effective for sense–antisense pairs, we evaluated its performance on antisense lncRNAs and their protein-coding counterparts, as well as on coding–coding pairs and non-coding–non-coding pairs. The results showed that the mean classification accuracy was 98% for coding–non-coding pairs, 87% for coding–coding pairs and 97% for non-coding–non-coding pairs, which was higher than that of CPC (95, 82 and 97%, respectively) and phyloCSF (63, 91 and 55%, respectively) (Supplementary Table S6, Supplementary Figure S8). These results demonstrated that CNCI tool is not only useful for classifying incomplete transcripts from RNA-Seq data but also has good performance of classifying sense–antisense transcript pairs.

### Application of CNCI to gene sets of multiple species and RNA-Seq data of poorly annotated species

Because gene annotation in multiple species (such as vertebrates, invertebrates and plants) has been partially completed by the Ensembl project (16), we tested CNCI on a series of species based on taxonomy. Interestingly, we found that using human data as training sets, CNCI performed well uniformly on all the species of the vertebrates (all MAE< 0.1), but relatively poorly on invertebrates and plants (MAE is 0.18 and 0.24, respectively) (Figure 2d). Although the accuracy and integrity of the known gene annotation varied across different species (i.e. human, mouse, *Caenorhabditis elegans* and *Arabidopsis thaliana* have higher quality of gene annotation than others), the distinct features of protein-coding and non-coding sequences between vertebrates, invertebrates and plants were obvious (Figure 2d). These results demonstrated that it is necessary to use invertebrates and plants as the training data to classify transcript sequences of the corresponding species, respectively. Our findings on the sequence characteristics may reflect changes in evolutionary trends of genes between species. Because RNA-Seq experiments have been carried out for many, although not well-studied, species,we tested CNCI performance on a published RNA-Seq data set from six organs of orangutan (3). Using the integrative approach to comprehensively reconstruct transcripts (11,17), we identified 110 154 expressed multiexonic transcripts, of which 88 563 (80%) had been annotated by Ensembl database, and 20 414 known genes, of which 13 678 corresponded to 67% known protein-coding genes. CNCI annotated 7697 novel transcripts as lncRNAs, including 631 intronic, 6029 intergenic and 1037 antisense RNAs that contributed to the orangutan lncRNA catalog (Supplementary data set 1). This can be applied to other species irrespective of the current annotation status.

## DISCUSSION

A large number of lncRNAs have been identified, facilitated by the rapid progress of high-throughput sequencing technology (11,18). Previous studies have demonstrated that lncRNAs are involved in diverse cellular processes, such as cell differentiation, imprinting control, immune responses, and a growing number of lncRNAs have been found to be implicated in disease etiology (19–21). However, for most species, it remains a challenge to identify lncRNAs from protein-coding genes because of the lack of necessary information such as whole-genome sequence, known protein database or conservative regions. Therefore, it is important to develop a method independent of known annotations to *de novo* classify lncRNAs and protein-coding genes. In this study, we found a powerful signature, the profile of the pairs of ANTs, which effectively distinguishes protein-coding or non-coding sequence regardless of species. Our finding was consistent with observations that the CDS regions have been under a variety of competing selection pressures, especially the translation optimization force that is associated with the juxtaposition of tRNAs but not required for non-coding regions (22). It is worth mentioning that a previous study used the length of the longest region in the transcript without stop codons to effectively discriminate the coding and non-coding sequences (23). This so-called stop-best feature was included in the ANT score matrix of our method. Similarly, a recent study demonstrated that the hexamer usage bias is a powerful indicator in the assessment of the protein-coding status of a sequence because of the sequence composition constraints introduced in the coding sequences by the genetic code (24). In addition, a gene finding program, GENSCAN, uses a homogeneous fifth-order Markov model for non-coding regions and an inhomogeneous fifth-order Markov model for coding regions of transcripts (25).

Although CNCI would be effective for classifying incomplete transcripts assembled from RNA-Seq data in most cases, caution should be taken in some cases. In mammalian genomes, at least 3′ exons of protein-coding transcripts may not extend significantly into the coding regions of transcripts. Instead, they may extend for several kilo base away, and occur abundantly in most of the RNA-Seq libraries, and thus are deemed as independent transcript units by most assembly tools. In such cases, CNCI may misclassify these 3′ (or 5′) partial sequences as non-coding RNAs; however, this misclassification is not because of the classification method *per se*, but the accuracy of the used assembly method. Therefore, the accurate query sets containing high-quality assembled transcripts are requisite to achieve optimal performance of CNCI.

CNCI is particularly well suited to the transcriptome analysis of the not well-studied species because it can effectively classify transcripts solely based on nucleotide composition of their sequence. The length of sequences we adopted in this work is >200 nt, and thus, theoretically, any sequence >200 bp can be analyzed using our proposed method. Our method differs from the previous methods that depend on information of known genome annotation or sequence conservation (4,5). Therefore, CNCI has a key advantage over other methods because genome sequences have been well annotated or completely sequenced only for limited species so far, and for most species, only partial or even none of their whole-genome sequences have been known. For these large number of species with poorly annotated sequences, it is hard to use peptide hits or multispecies alignments to classify sequences into protein-coding or non-coding transcripts, as different ORF cutoffs may lead to a high false-negative/positive rate, especially for lncRNAs (7). Although sequence search approaches for the discrimination between protein-coding and non-coding transcripts have been available (26), there is still lack of effective *de novo* approach to achieve it. Thus, CNCI is a useful tool, not only for predicting protein-coding or non-coding sequences for high-throughput sequencing data of numerous species but also for analyzing the sequence features across species as a way to gain insights into the evolution.

## SUPPLEMENTARY DATA

Supplementary Data are available at NAR Online.

## FUNDING

Training Program of the Major Research plan of the National Natural Science Foundation of China (91229120); International Science and Technology Cooperation Projects (2010DFA31840 and 2010DFB33720). Funding for open access charge: Training Program of the Major Research plan of the National Natural Science Foundation of China [91229120].

*Conflict of interest statement.* None declared. 

## Supplementary Material

Supplementary Data
