# Supplementary material for: Utilizing sequence intrinsic composition to classify protein-coding and long non-coding transcripts
Source: Nucleic Acids Res. 2013 Jul 27;41(17):e166. doi: 10.1093/nar/gkt646 (PMC3783192; doi:10.1093/nar/gkt646)
Supplement: Supplementary Data [file supp_41_17_e166__index.html]

Utilizing sequence intrinsic composition to classify protein-coding and long non-coding transcripts — Utilizing sequence intrinsic composition to classify protein-coding and long non-coding transcripts — Utilizing sequence intrinsic composition to classify protein-coding and long non-coding transcripts — Supplementary Data 

# Utilizing sequence intrinsic composition to classify protein-coding and long non-coding transcripts

## 

files

**Files in this Data Supplement:**

- Supplementary Data - pdf file
- Supplementary Data - xls file
- Supplementary Data - xls file
