# Supplementary material for: Utilizing sequence intrinsic composition to classify protein-coding and long non-coding transcripts
Source: Nucleic Acids Res. 2013 Jul 27;41(17):e166. doi: 10.1093/nar/gkt646 (PMC3783192; doi:10.1093/nar/gkt646)
Supplement: Supplementary Data [file supp_gkt646_nar-01516-met-z-2013-File004.pdf]

Supplementary Materials

Supplementary methods

The method described in this work has been assembled as a Perl package, i.e., CNCI, and is available for download at http://www.bioinfo.org/software/cnci. This package is distributed with a tutorial that will enable the user to reproduce the type of analysis we present in this work.

Comparison of CNCI performance with CPC and phyloCSF

To compare CNCI performance with other methods, we re-analyzed the testing set using CPC and phyloCSF. To run CPC analyses, we submitted the testing transcripts to CPC's website directly in Fasta format and used default parameters, and a list of definitive results marked with protein-coding and non-coding following the quality score were returned. To perform phyloCSF analyses, we uploaded the testing transcripts in BED format to the Galaxy, which converted the BED file to 29 species multiple alignment format by "stitch MAF blocks" function. After that, we submitted these multiple alignment files to phyloCSF (downloaded from http://compbio.mit.edu/PhyloCSF) with the parameter --minCodons=30, --orf=ATGStop, --strategy=fixed, --frames=3, and --removeRefGaps. We then classified these transcripts according to phyloCSF's results (if the score of one transcript > 0, we considered it as a coding transcript, otherwise as a non-coding transcript).

Performance assessment

In a cross-validation process, the training-set was divided into 10 individuals randomly, a classifier was trained on nine of these ten. Next, we assessed the performance using the remaining one and repeated such a process 10 times. After that, we plotted the data to the sensitivity-specificity curves for classification on the testing set. In particular, we utilized 14353 human lincRNAs to determine the association between the size of noncoding transcripts and performance accuracy. These lincRNAs were divided into several different sets based on their length, and length-span (i.e., 200-300, 300-500,500-1000, 1000-1500, 1500-2000 and >2000 bp). For each set, the classification results were calculated by CNCI, CPC, and phyloCSF, respectively.

In order to assess our performance on incomplete transcripts, we willingly removed the exon in 3' or 5' end from all multiexonic transcripts (exon number>3) and made sure that the remaining CDS of protein-coding transcripts were more than fifty percent of the entire CDS. We then analyzed them accordingly.

**Supplementary files**	**Description**
**Figure S1**	The profiles of usage frequency of ANT
**Figure S2**	Robust test of parameter N
**Figure S3**	Distribution of overlap degree between MLCDS and CDS
**Figure S4**	Score distribution between coding and noncoding sequences for the first four features selected to build the SVM model.
**Figure S5**	The log-ratio of the usage frequency between protein-coding and non-coding sequences for each nucleotide triplet
**Figure S6**	Distribution of S-score
**Figure S7**	Distribution of these six reading frames of protein-coding and non-coding transcripts
**Figure S8**	The ROC analyses of CNCI for classification of sense-antisense pairs
**Table S1**	Description of datasets
**Table S2**	Length distribution of the human and mouse transcript collections
**Table S3**	Significance of features
**Table S4**	Information about the trimming process
**Table S5**	Compare CNCI with CPC and phyloCSF in incomplete transcripts
**Table S6**	CNCI performance on sense-antisense pairs
**Supplementary dataset 1**	The bed file of orangutan long non-coding RNA

Note: Table S6 and Supplementary dataset 1 are uploaded separately.

Supplementary Figures

Figure S1. Profiles of usage frequency of ANT.

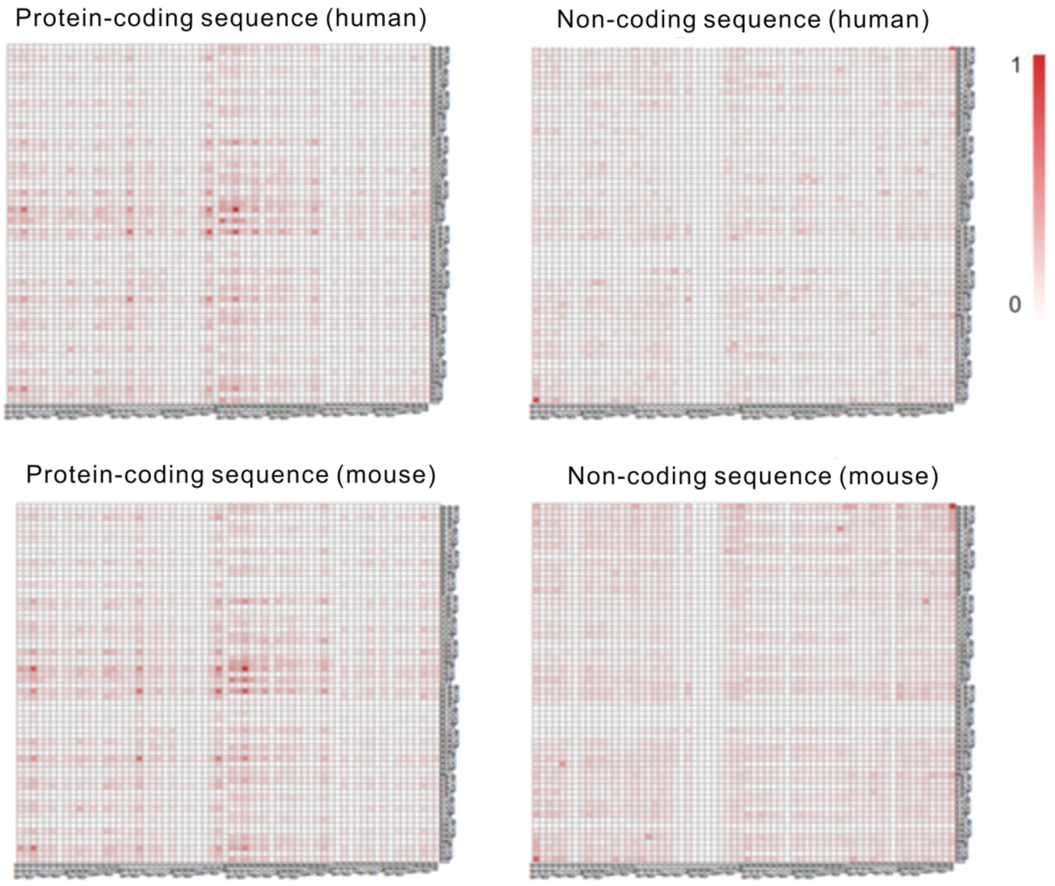

Figure S1. Profiles of usage frequency of ANT. A total of six 64*64 matrices are shown of the usage frequency of ANT in human or mouse protein-coding and non-coding sequences. The Pearson's correlation test shows a p value < 0.001 between human and mouse protein-coding transcripts (r = 0.986538); p value < 0.001 between human protein-coding and non-coding transcripts (r = 0.455); p value < 0.001 between mouse protein-coding and non-coding transcripts (r = 0.40612).

Figure S2. Robust test of the parameter N.

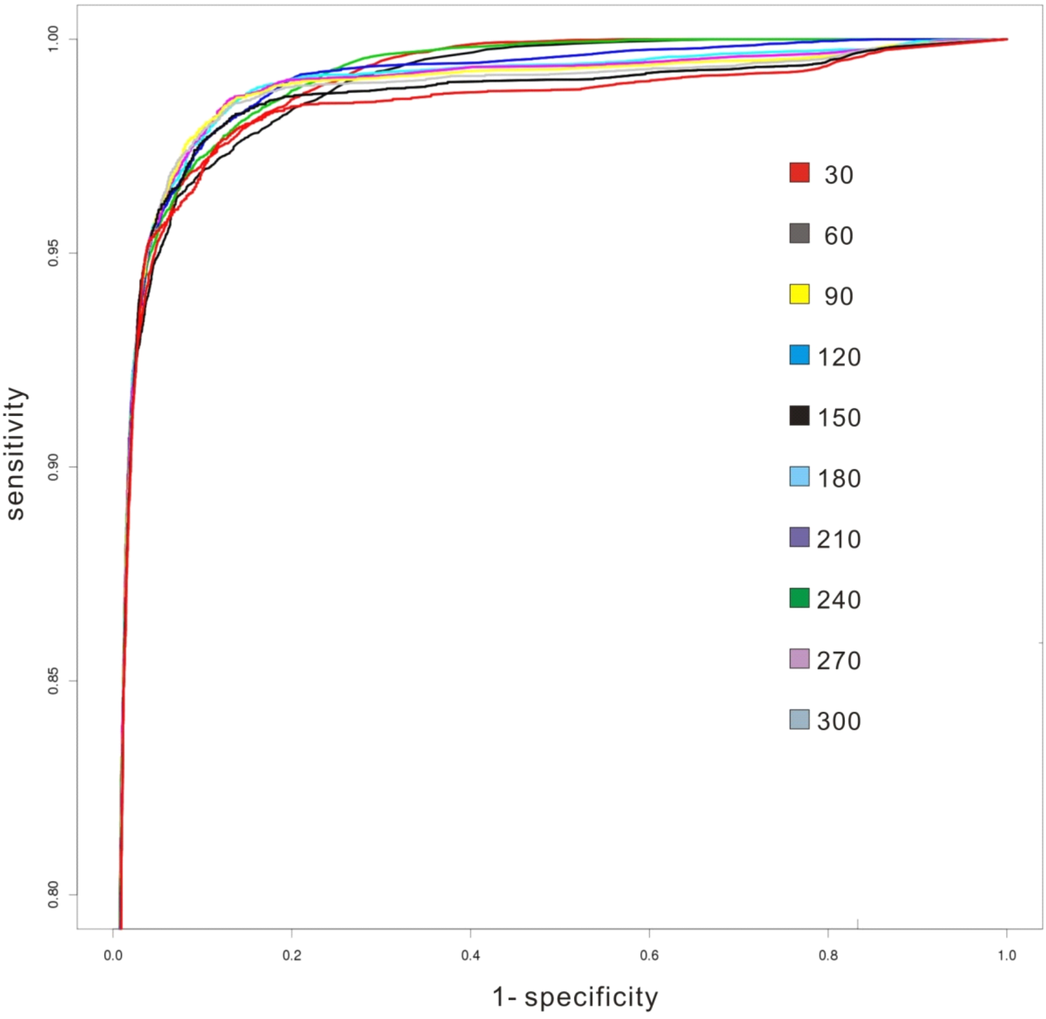

Figure S2. Robust test of the parameter N. Sensitivity-specificity curves with different lengths (N from 30nt to 300nt with 30nt as a step).

Figure S3. Distribution of overlap degree between MLCDS and CDS.

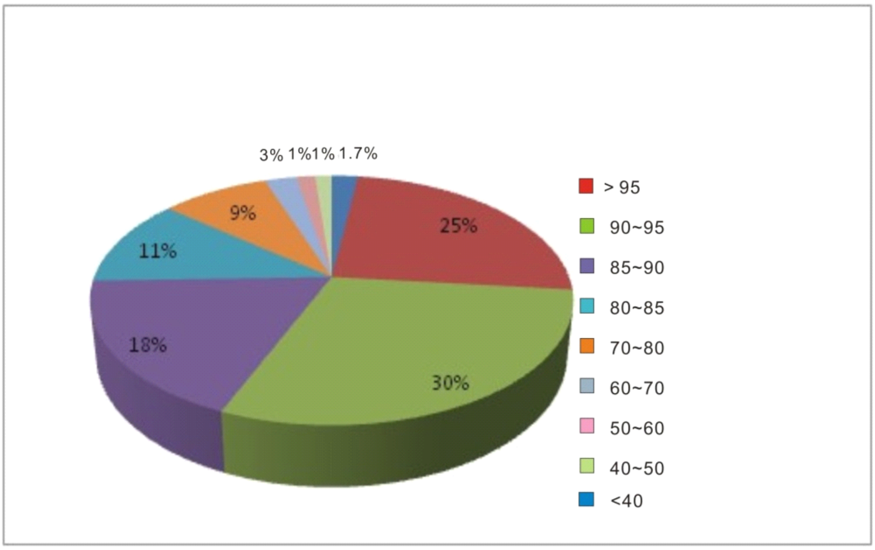

Figure S3. Distribution of overlap degree between MLCDS and CDS. The overlap degree between MLCDS and true CDS are separated into nine intervals indicated by different colors. The numbers marked on pie represent the percentage of transcripts that located in the intervals of corresponding overlap degree.

Figure S4. Score distribution between coding and noncoding sequences for the first four features selected to build the SVM model.

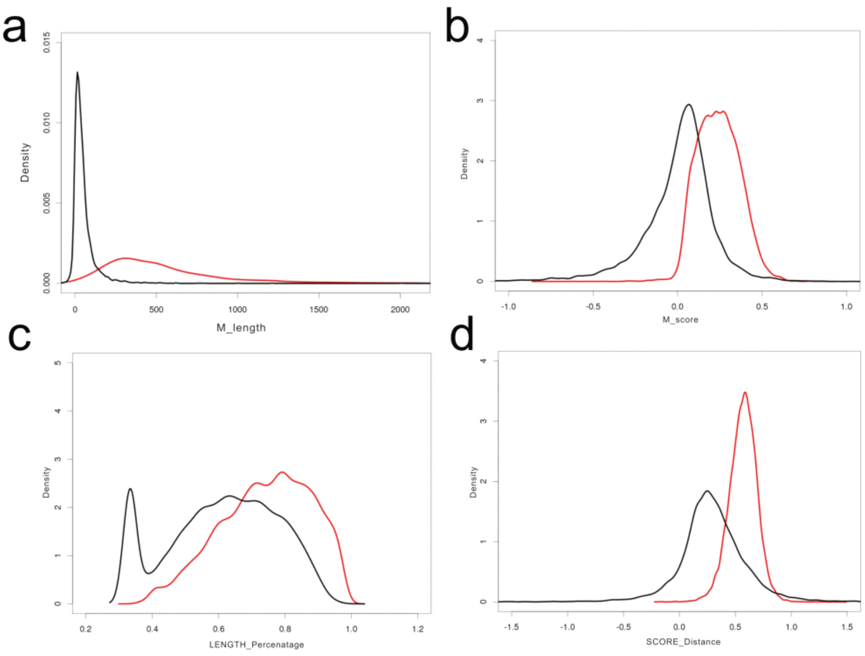

Figure S4. Score distribution between coding (red) and non-coding (black) sequences for the first

four features selected to build the SVM model; human protein-coding gene and long noncoding transcript sets were used. (a) S-score of MLCDS. (b) Length of MLCDS. (c) Length-percentage (c) and Score-distance (d) of MLCDS.

Figure S5. The log-ratio of the usage frequency between protein-coding and non-coding sequences for each nucleotide triplet

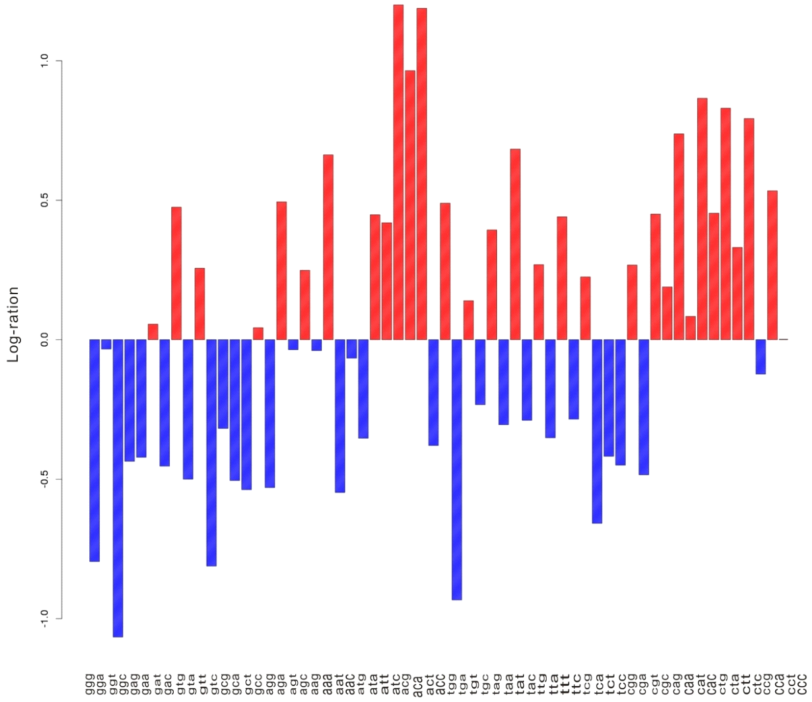

Figure S5. The log-ratio of the usage frequency between protein-coding and non-coding sequences for each nucleotide triplet. The X-axis is the list of 61 nucleotide triplets (ruled out three stop codons), whereas the Y-axis represents the log-ration of the usage frequency. If the usage frequency of a nucleotide triplet is more than 0, it would indicate positive protein-coding potential and marked in red. In contrast, if the usage frequency of a nucleotide triplet is less than 0, it would indicate negative protein-coding potential and marked in blue.

Figure S6. Distribution of S-score

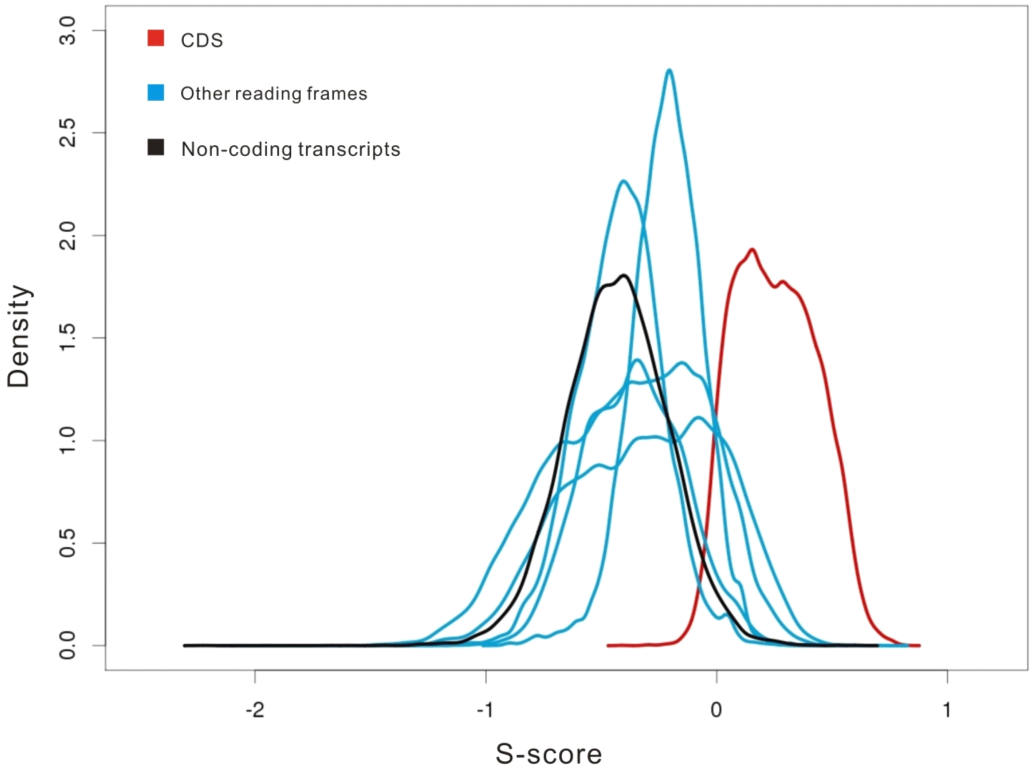

Figure S6. Distribution of S-score. The red line represents the S-sore distribution of true CDS; the blue lines represent the S-sore distribution of other five reading frames of protein-coding transcripts; the black line represents the S-sore distribution of human non-coding transcript.

Figure S7. Distribution of these six reading frames of protein-coding and non-coding transcripts

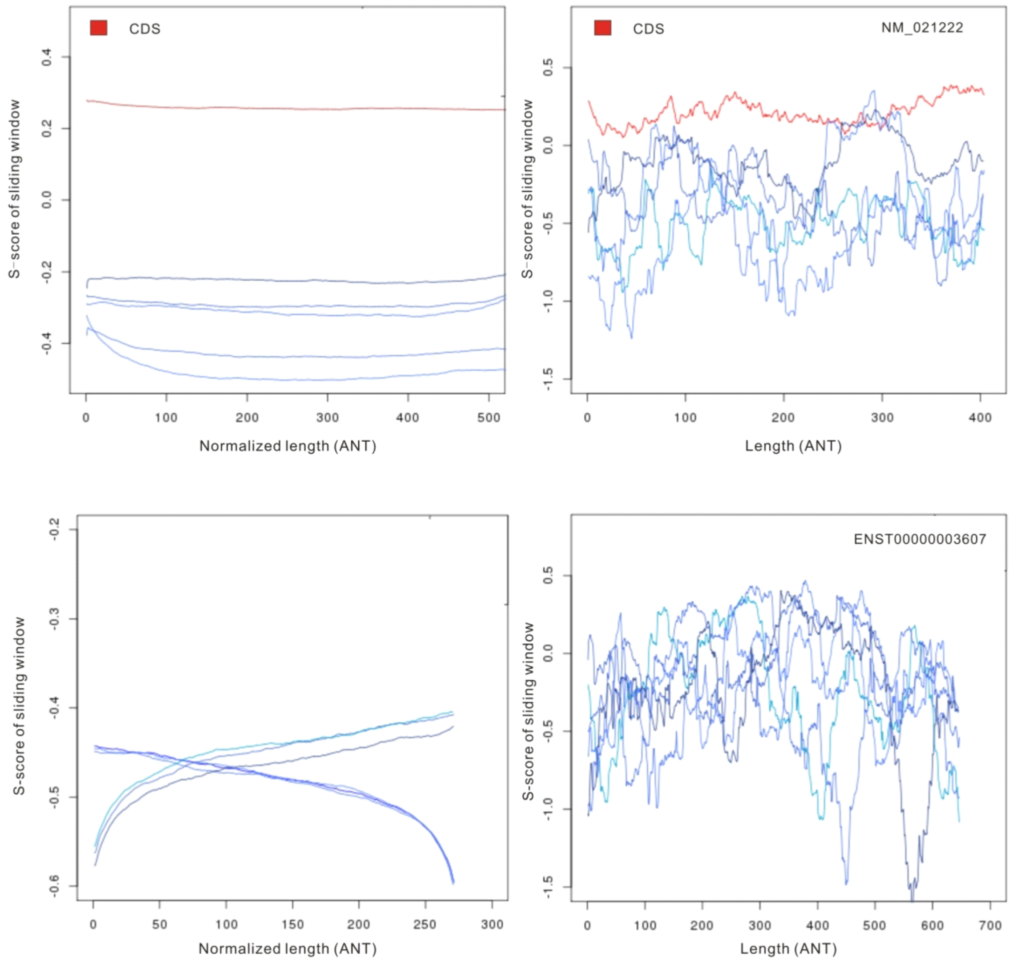

Figure S7. Distribution of these six reading frames of protein-coding (top-left) and non-coding (bottom-left) transcripts. Examples are shown in the top-right (NM_021222) and the bottom-right (ENST00000003607). In the top panels, the red line represents the true CDS and other five blue lines represent the other five reading frames of protein-coding transcripts. In the bottom panels, a series of blue lines represent these six reading frames of non-coding transcripts. The X-axis represents the length (or normalized length) of the transcripts in ANT format (1 ANT=3 nt), whereas the Y-axis represents the S-score of each sliding window. The length of sequence is normalized into 0-500 nucleotide triplets for protein-coding transcripts and 0-300 for non-coding transcripts.

Figure S8. The ROC analyses of CNCI for classification of sense-antisense pairs

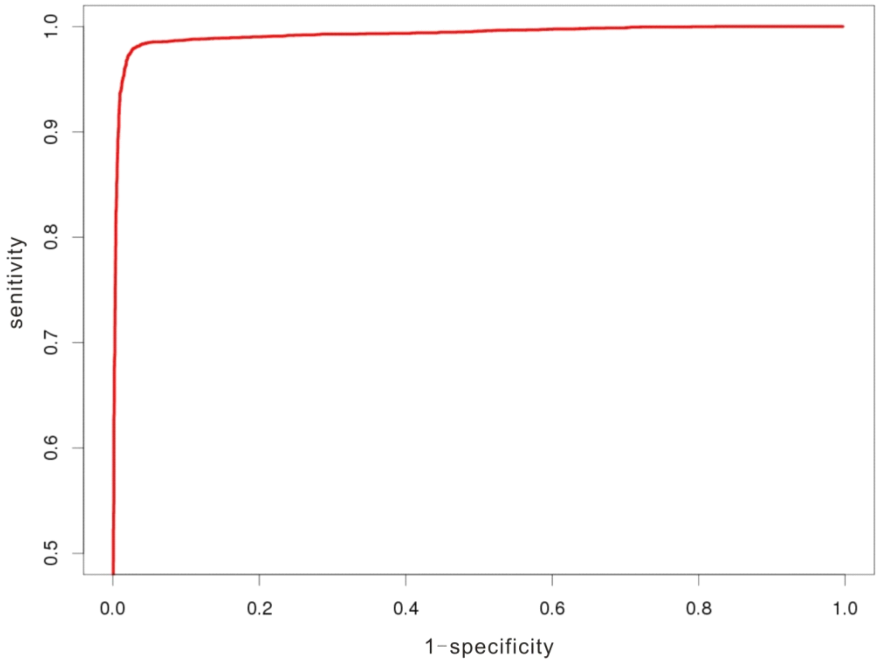

Figure S8. The ROC analyses of CNCI for classification of sense-antisense pairs. The accuracy is 97.8% and 98% for antisense noncoding RNAs and their coding counterparts respectively.

Table S1. Description of datasets

Species	Datasets	Number of protein-coding transcripts	Number of non-coding transcripts
Human	RefSeq	30507	NA
Human	Gencode.v11	NA	18566
Human	Human Body Map	NA	14353
Mouse	RefSeq	25316	NA
Mouse	Esembel (v69)	NA	8696
Anole lizard	Esembel (v69)	4667	3136
Chicken	Esembel (v69)	21094	1102
Gorilla	Esembel (v69)	26295	6701
Xenopus	Esembel (v69)	4352	1282
Macaque	Esembel (v69)	34721	6579
Chimpanzee	Esembel (v69)	19329	8657
Lamprey	Esembel (v69)	10725	2628
Orangutan	Esembel (v69)	20177	6996
C.elegans	Esembel (v69)	30372	23876
Zebrafish	Esembel (v69)	4554	4431
Arabidopsis	Esembel (v69)	34119	480

Table S2. Length distribution of the human and mouse transcript collections

Note: the value in the table represents the number of transcripts located in the different length intervals

		200-300nt	300-500nt	500-1000 nt	1000-1500 nt	1500-2000 nt	>2000nt
Protein-coding transcripts	RefSeq (for human)	0	12	1275	3151	4258	21811
	RefSeq (for mouse)	741	338	3428	3248	3393	16055
Non-coding transcripts	Gencode	882	4823	8542	1141	994	2184
	Human Body Map	1729	4150	4795	1354	821	1504
	Ensembl	378	568	1058	484	368	819

Table S3. Significance of features

Species	Datasets	Index	Average of M-score	Average of M-length	Average of Length-percentage	Average of Score-distribution
Human	Refseq	Coding	145.4	1641	61.68%	137.80
Human	Gencode	Non-coding	2.2287	120	33.3%	2.841
Mouse	Refseq	Coding	136.318	1638	68.18%	132.63
Mouse	Ensembl	Non-coding	-1.29	117	25%	10.43

Table S4. Information about the trimming process

Trimming direction	Average length of removed sequence (nt)	Protein-coding transcripts with whole CDS after trimming	Average length of CDS before trimming (nt)	Average length of CDS after trimming (nt)
Trimming the first exon from 5’end	980	28.3%	1806	1230
Trimming the first exon from 3’end	970	45.2%	1806	1580

Table S5. Compare CNCI with CPC and phyloCSF in incomplete transcripts

Method	Cutting set	Average accuracy	Accuracy for coding sequence	Accuracy for non-coding sequence
CNCI	5`end	97.7%	97.1%	98.2%
	3`end	97.9%	97.5%	98.3%
CPC	5`end	87.9%	98.6%	77.2%
	3`end	87.1%	99.0%	75.1%
phyloCSF	5`end	82.3%	96.2%	68.4%
	3`end	82.0%	96.5%	67.5%
